# Bifunctional nanodisc platforms for studies on self-assembly and amyloid fibrillation†

**DOI:** 10.1039/d5cc00972c

**Published:** 2025-07-08

**Authors:** Bikash R. Sahoo, Takahiro Watanabe-Nakayama, Saba Suladze, G. M. Anantharamaiah, Bernd Reif, Ayyalusamy Ramamoorthy

**Affiliations:** a Biophysics, Department of Chemistry, University of Michigan Ann Arbor MI 48109 USA bsahoo@umich.edu; b WPI-Nano Life Science Institute, Kanazawa University Kanazawa 9201192 Japan; c Department of Chemistry, Technische Universität München Garching 85748 Germany; d Department of Medicine, UAB Medical Center Birmingham AL 35294 USA; e Department of Chemical and Biomedical Engineering, FAMU-FSU College of Engineerig, National High Magnetic Field Laboratory, Institute of Molecular Biophysics, Florida State University Tallahassee FL 32317 USA aramamoorthy@fsu.edu

## Abstract

Nanodiscs are emerging as valuable tools for studying lipid–protein interactions. In this study, we demonstrate the dual role of nanodiscs in modulating human amylin (hIAPP) fibrillation using NMR and HS-AFM. Peptide-based nanodiscs promote hIAPP fibrillation, while polymer-based nanodiscs inhibit aggregation. These findings broaden the applications of nanodiscs beyond structural studies.

## Abstract 2

Modulating human amylin aggregation with nanodiscs.

## Graphical abstract

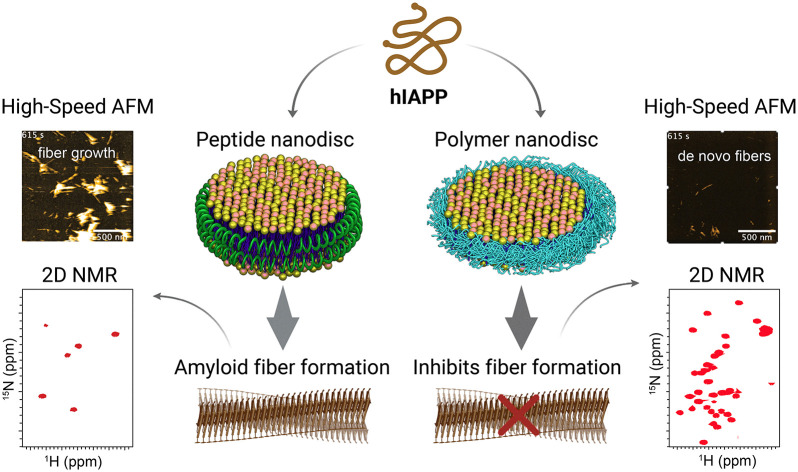

Protein-conformational diseases are linked to various neuropathic and non-neuropathic conditions, including type-2 diabetes (T2D).^1^ During the progression of these diseases, misfolded proteins aggregate to form insoluble structures known as amyloids,^2^ which contribute to disease advancement through cellular degeneration. Amylin, also known as human islet amyloid polypeptide (hIAPP), is an intrinsically disordered 37-residue peptide hormone, and its fibrillation is associated with T2D.^3^ hIAPP is an endocrine partner to insulin and is co-secreted with insulin in the pancreas.^4^ For reasons not yet fully understood, disruptions in cellular balance alter hIAPP-to-insulin ratio, leading to hIAPP fibrillation.

hIAPP aggregation is a critical factor in T2D,^5^ contributing to β-cell dysfunction and pancreatic islet failure. While multiple factors influence hIAPP fibrillation, lipid membranes^6,7^ play a particularly important role in modulating fibrillation kinetics and toxicity. Controlling hIAPP fibrillation within cells and understanding the role of the cell membrane are of significant interest. hIAPP aggregation at the membrane interface,^8^ its membrane permeabilization,^9^ and the impact on β-cell dysfunction are important to explore.^10^ However, high-resolution, real-time studies of hIAPP fibrillation at the membrane interface are challenging. Here, we use nanodiscs as a biomimetic platform to investigate how the lipid environment influences amyloid fibrillation in real-time using NMR, high-speed atomic force microscopy (HS-AFM), and molecular dynamics (MD) simulations. Nanodiscs are emerging as a valuable tool for investigating the early phases of amyloid fibrillation and have been shown to be useful in trapping amyloid intermediates for structural characterization.^11,12^ Our findings provide insight into physiologically relevant mechanisms that may regulate hIAPP assembly in pancreatic β-cells and offer potential applications of nanodiscs in targeting membrane interactions for therapeutic intervention.

The effects of two different types of nanodiscs, polymer (PMA)^13^ or peptide (4F)^14^ encapsulated, on amyloid fibrillation are investigated in this study. A 4 : 1 DMPC : DMPG molar ratio was used in these nanodiscs (Fig. 1a) that are monodispersed with an average size of ∼10 nm diameter, as determined by dynamic light scattering (DLS), which relates to the size observed in HS-AFM (Fig. S1, ESI†). The effects of these nanodiscs on hIAPP fibrillation were then measured by a thioflavin-T (ThT) fluorescence assay. As shown in Fig. 1b and Fig. S2 (ESI†), the presence of 4F-nanodiscs reduced the half-time of hIAPP aggregation as well as the ThT intensity as compared to hIAPP alone. These observations are in agreement with anionic membranes promoting hIAPP fibrillation and altering fibril morphology, as reported in the literature.^8^ On the other hand, the presence of PMA-nanodiscs exhibited >10 hours half-time at a 1 : 2 hIAPP : nanodiscs, and no significant fibril formation for 1 : 20 and 1 : 100 hIAPP : nanodiscs samples even after 24 hours (Fig. 1c and Fig. S2, ESI†).

**Fig. 1:**
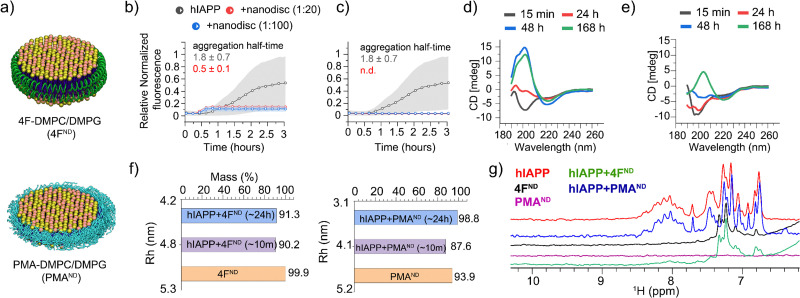
(a) Schematic representation of 4F- and PMA-nanodiscs. (b) and (c) ThT fluorescence assays show that 4F-nanodiscs accelerate hIAPP aggregation (half-time 0.5 ± 0.1 h) (b), while PMA-nanodiscs inhibit it (c). 20-hour kinetics data are shown in Fig. S2 (ESI†). Gray background curves represent the standard deviation from three independent control measurements of 5 μM hIAPP aggregation in the absence of nanodiscs. (d) and (e) CD spectra of hIAPP alone (d) and hIAPP with 1 : 20 PMA-nanodiscs (e), illustrating delayed fibrillation in nanodisc-containing samples. (f) DLS measurements tracking nanodisc size changes upon hIAPP binding. (g) Amide-proton NMR spectra showing significant line broadening of hIAPP peaks in the presence of 4F-nanodiscs (green), indicating hIAPP aggregation, while no significant changes were observed with PMA-nanodiscs (blue).

These nanodisc-belt dependent effects were next investigated using CD and 1D proton NMR. CD data showed PMA-nanodiscs to be stabilizing the initial random-coil-rich structure of hIAPP for ∼24 hours, resulting in a β-structure at ∼168 hours (Fig. 1e), whereas hIAPP alone forms a β-structure corresponding to matured fibrils at ∼48 hours (Fig. 1d). NMR experiments were performed to analyze the amide-proton chemical shift dispersion (∼7 to 8.5 ppm region) as a narrow dispersion may be correlated to unstructured hIAPP, and line broadening can be an indication of hIAPP aggregation. It should be noted that signals from 4F-nanodiscs (Fig. 1g, black) do not overlap with the amide (N–H) peaks from hIAPP, while PMA-nanodiscs showed no peaks (purple) in the region of interest (Fig. 1g). Interestingly, hIAPP mixed with or without PMA-nanodiscs showed similar spectra suggesting no significant structural change for hIAPP (Fig. 1g, blue and red), which correlates with our fluorescence and CD results. On the contrary, hIAPP mixed with 4F-nanodiscs showed undetectable N–H proton signals (Fig. 1g, green), indicating hIAPP fibrillation as observed in the fluorescence assay. We next set out to probe the nanodisc integrity and shape, as hIAPP binding can affect the structure of the lipid bilayer. DLS measurements estimated a hydrodynamic diameter of ∼10 nm for both 4F- and PMA-nanodiscs before hIAPP addition. Whereas a ∼24-hour incubation of hIAPP in nanodiscs showed a substantial size reduction: ∼7 and ∼8.6 nm in hydrodynamic diameter for PMA- and 4F-nanodiscs, respectively (Fig. 1f), suggesting hIAPP affects nanodisc size. Other than the decrease in nanodisc size and PMA's tendency to directly interact with hIAPP,^13^ the exact reasons for the reduction in the nanodisc size are not clear. Nevertheless, the DLS data indicated the existence of lipid bilayer nanodiscs even after ∼24 hours.

A handful of studies have demonstrated the application of nanodiscs in characterizing structures of amyloid intermediates.^11,12,15^ Our observation of the dual function of nanodiscs motivated us to further investigate their effects using HS-AFM.^16^ This method has previously been shown to track amyloid fibril growth in real-time in the presence and absence of ions, polymers, nanoparticles, and other substances.^17^ Both 4F- and PMA-nanodiscs, when deposited on a mica surface without hIAPP, displayed spherical and well-dispersed particles with a size of approximately 10 nm (Fig. 2a, b, and Movies SV1, SV2, ESI†). The addition of freshly prepared hIAPP monomers (1 or 5 μM) filled the space between PMA-nanodiscs (Fig. S3, and Movies SV3–SV8, ESI†) on the HS-AFM stage within ∼10 minutes (Fig. 2c and d). Additionally, no hIAPP fibers were observed in either of the nanodisc samples tested, despite using different peptide concentrations ranging from 200 nM to 5 μM (Fig. 2c, d, and Fig. S3, ESI†). This observation is consistent with fluorescence results obtained from PMA-nanodiscs, but inconsistent with those from 4F-nanodiscs, for which fluorescence assay revealed hIAPP fibrillation (Fig. 1b and c). This apparent discrepancy between ThT fluorescence and HS-AFM observations regarding fibril formation in 4F-nanodiscs may be attributed to the differences in experimental conditions and the inherent limitations of surface-bound techniques. The immobilization of nanodiscs on mica during HS-AFM imaging could restrict fibril growth and dynamics, potentially leading to an underestimation of fibril formation. Additionally, the presence of nanodiscs on the mica surface precludes an exposed mica surface necessary for hIAPP fibrils to adhere.^18^

**Fig. 2:**
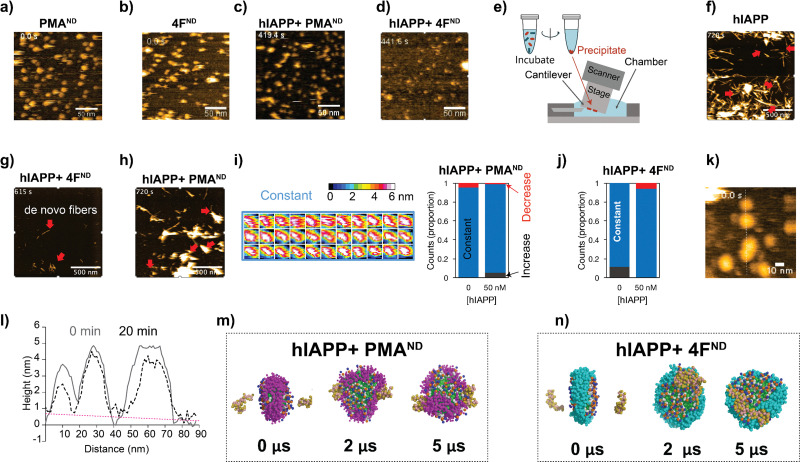
Real-time visualization of nanodisc-mediated modulation of hIAPP fibrillation using HS-AFM and MD simulations. (a) and (b) HS-AFM images of PMA- (a) and 4F-nanodiscs (b) alone, without hIAPP. (c) and (d) PMA- (c) and 4F-nanodiscs (d) incubated with 0.5 μM hIAPP in 30 mM NaOAc, pH 5.5, show no detectable fibril formation, suggesting suppression of early aggregation. (e) Schematic of the experimental workflow used to track fibril growth after nanodisc preincubation, followed by deposition onto mica for HS-AFM imaging. (f)–(h) Time-lapse HS-AFM images showing fibril elongation (red arrows) during incubation of preformed hIAPP fibrils with 5 μM monomeric hIAPP: (f) without nanodiscs (uncontrolled growth), (g) with 4F-nanodiscs, and (h) with PMA-nanodiscs. (i) and (j) Heatmap analyses indicate minimal nanodisc morphological changes in nanodiscs upon binding to 50 nM hIAPP. Nanodiscs showing no change are marked in blue, increased thickness in gray, and reduced thickness in red, highlighting differential interactions of PMA- (i) and 4F-nanodiscs (j) with hIAPP. (k) Height profile analysis of 4F-nanodiscs along the dashed line shows changes in morphology due to hIAPP monomer binding at 0 and 20 minutes. (l) The dashed line indicates a negative slope in the background. (m) and (n) Coarse-grained MD simulations show atomic-level interactions of two hIAPP monomers (orange) with self-assembled PMA- (purple, m) and 4F- (cyan, n) nanodiscs; lipid tails are shown in green. Snapshots represent selected timepoints from a 5 μs simulation, illustrating distinct nanodisc-hIAPP binding interfaces.

We then investigated the effect of nanodisc binding on the structure of hIAPP fibrils by incubating aged fibrils with or without nanodiscs for ∼24 hours, followed by precipitation, resuspension, and deposition onto a mica surface (Fig. 2e–h, see Methods). In samples incubated with hIAPP fibrils without nanodiscs, we observed typical fibril structures (Fig. S4, upper right, ESI†). When hIAPP monomer was added, fibril elongation occurred (Fig. 2f and Movie SV9, ESI†), confirming the stability of the hIAPP fibrils. Similarly, fibrillar structures were present in samples where hIAPP fibrils were mixed with PMA-nanodiscs (Fig. S4, upper middle, ESI†). The addition of hIAPP monomer led to further fibril elongation (Fig. 2h and Movie SV11, ESI†), indicating that the fibrils remained relatively stable in the presence of PMA-nanodiscs. However, when hIAPP fibrils were mixed with 4F-nanodiscs, the resulting precipitates showed only thin (∼2 nm) and short fibers under HS-AFM (Fig. S4, upper left, ESI†), in contrast to the typical fibrillar structure. These thin fibers were also present in precipitates from 4F-nanodiscs alone, without hIAPP fibers (Fig. S4, lower left, ESI†). These results suggest that 4F-nanodiscs interact with and dissociate hIAPP fibrils because images of hIAPP fibrils disappeared during incubation with 4F-nanodiscs (Fig. 2g). Moreover, the addition of hIAPP monomer led to the formation of *de novo* fibrils, further indicating that the dissociated fibers do not seed fibril formation (Fig. 2g and Movie SV10, ESI†).

Given the role of PMA polymers in binding and inhibiting hIAPP fibrillation,^13^ we speculate that the irregular nanodisc shape, reduced nanodisc density, and the presence of non-fibrillary hIAPP species observed in the absence of preformed hIAPP fibers could be attributed to differential hIAPP deposition on the nanodisc surface. To investigate this, we analyzed nanodisc height as a proxy for size, as hIAPP filling prevented direct measurements of the nanodiscs in the *XY* plane (Fig. S3, ESI†). The height profile analysis, indicative of membrane thickness in nanodiscs binding to 50 nM hIAPP, showed minimal to no change (Fig. 2i and j). 4F-nanodiscs incubated with 1.5 μM hIAPP exhibited slight variations in size (Fig. 2k). Single PMA-nanodisc analysis further demonstrated that hIAPP had a minimal impact on membrane thickness, with an average height of approximately 4 nm (Fig. 2i). Cross-sectional analysis of 4F-nanodiscs before and 20 minutes after hIAPP addition revealed negligible changes in nanodisc height, as indicated by the flat slope in the background (Fig. 2i). Real-time HS-AFM observations suggest that hIAPP did not significantly alter membrane thickness in both types of nanodiscs, indicating that nanodiscs could trap hIAPP in low-ordered intermediate states. It is important to note that one of the two surfaces of the nanodisc interacting with the HS-AFM stage is not accessible to hIAPP, which may affect hIAPP's natural interaction with lipids.

To further explore the binding and assembly of hIAPP on nanodiscs at high resolution, coarse-grained MD simulations were carried out to monitor how hIAPP interacts with nanodiscs. Over a 5 μs timeframe, simulations revealed hIAPP monomers initially positioned on opposite faces of discoidal-shaped PMA-nanodiscs to be primarily interacting with the PMA belt (Fig. 2m). In contrast, hIAPP molecules tended to form dimeric species on the lipid bilayer surface of 4F-nanodiscs (Fig. 2n). These findings indicate that PMA-nanodiscs effectively trap and stabilize hIAPP monomers, whereas 4F-nanodiscs promote hIAPP oligomerization and aggregation.

To understand the distinct roles of PMA- and 4F-nanodiscs in modulating hIAPP aggregation at the residue level, 2D ^15^N/^1^H NMR experiments were carried out. NMR spectra (Fig. 3) revealed a monomer-like distribution of resonances for hIAPP mixed with PMA-nanodiscs, whereas a significant loss of peaks for hIAPP mixed with 4F-nanodiscs (Fig. 3). Despite insignificant chemical shift perturbations (Fig. S5 and S6, ESI†), the presence of PMA-nanodiscs exhibited slightly higher peak volume for residues in the central region of hIAPP, suggesting that the nanodiscs either stabilize the monomeric or soluble oligomeric form of hIAPP, which could impede the peptide aggregation. The insignificant chemical shift perturbation suggests that PMA-nanodiscs and hIAPP interaction is dynamic and occurs without major structural rearrangement, which is in agreement with CD results. In contrast, 4F-nanodiscs lead to substantial peak intensity loss for most residues (Fig. 3), suggesting enhanced aggregation or sequestration of hIAPP into large, NMR-invisible complexes. These observations are consistent with fluorescence, CD, and MD simulations results, which indicate that PMA-nanodiscs preferentially bind with monomeric hIAPP at the polymer belt, while 4F-nanodiscs facilitate peptide self-association on the lipid bilayer (Fig. 2m and n). Thus, PMA-nanodiscs stabilize disordered hIAPP species, whereas 4F-nanodiscs promote fibril nucleation. Further studies are required to assess the impact of nanodisc size and lipid composition on hIAPP aggregation.

**Fig. 3:**
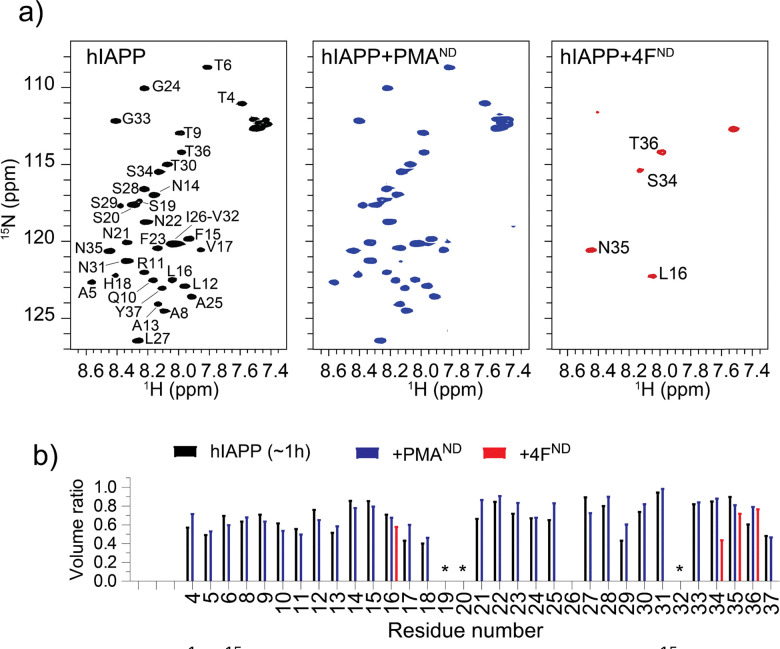
(a) 2D ^1^H/^15^N SOFAST-HMQC spectra of 30 μM ^15^N-labeled hIAPP monomers in the absence and presence of nanodiscs containing 250 μM DMPC/DMPG (4 : 1) lipids. Spectra were recorded approximately 1, 1.5, and 2 hours after sample preparation for hIAPP alone, hIAPP + PMA nanodiscs, and hIAPP + 4F nanodiscs, respectively. (b) Peak volume ratios measured from the 2D spectra shown in (a), normalized to freshly prepared hIAPP. Overlapping residues were excluded from analysis and marked with an asterisk (*).

In conclusion, this study demonstrates that nanodiscs can modulate amyloid fibrillation, with effects tunable *via* nanodisc belt chemistry. The ability of PMA-nanodiscs to trap misfolded hIAPP monomers enables real-time probing of their dynamics. Conversely, 4F-nanodiscs, which promote hIAPP–lipid interactions, may help to elucidate the role of lipids in amyloid fibrillation.

B. R. S. and A. R. conceived the idea; S. S. and B. R. generated hIAPP, and G. M. A. generated 4F peptides. A. R. supervised the research and obtained funding; B. R. S. and T. W.-N. performed experiments and data analysis; B. R. S., T. W.-N. and A. R. interpreted the results. B. R. S. and A. R. wrote the manuscript, and all authors approved the manuscript.

This study was supported by funding from the NIH (R01DK132214 to A. R.) and World Premier International Research Center Initiative (WPI), MEXT, Japan.

## Conflicts of interest

PMA is patented.^19^

## Supplementary Material

CC-061-D5CC00972C-s001

CC-061-D5CC00972C-s002

CC-061-D5CC00972C-s003

CC-061-D5CC00972C-s004

CC-061-D5CC00972C-s005

CC-061-D5CC00972C-s006

CC-061-D5CC00972C-s007

CC-061-D5CC00972C-s008

CC-061-D5CC00972C-s009

CC-061-D5CC00972C-s010

CC-061-D5CC00972C-s011

CC-061-D5CC00972C-s012

## Data Availability

The data supporting this article have been included in the ESI.†
