# Supplementary material for: Bifunctional nanodisc platforms for studies on self-assembly and amyloid fibrillation
Source: Chem Commun (Camb). 2025 Jul 8;61(66):12349–52. doi: 10.1039/d5cc00972c (PMC12264740; doi:10.1039/d5cc00972c)
Supplement: CC-061-D5CC00972C-s001 [file CC-061-D5CC00972C-s001.pdf]

Supplementary Information (SI) for ChemComm.
This journal is © The Royal Society of Chemistry 2025

# Supporting information

## Bifunctional Nanodisc Platforms for Studies on Self-Assembly and Amyloid Fibrillation

Bikash R. Sahoo,[*a] Takahiro Watanabe-Nakayama,[b] Saba Suladze,[c] GM Anantharamaiah,[d] Bernd Reif,[c] and Ayyalusamy Ramamoorthy[*a,e]

[a]Biophysics and Department of Chemistry, University of Michigan, Ann Arbor, MI 48109, USA.

[b]Bio-AFM Frontier Research Center, Kanazawa University, Kanazawa 9201192, Japan.

[c]Department of Chemistry, Technische Universität München, Garching 85748, Germany

[d]Department of Medicine, UAB Medical Center, Birmingham, AL 35294, USA

[e]Department of Chemical and Biomedical Engineering, National High Magnetic Field Laboratory, Florida State University, Tallahassee, FL 32317, USA

*Corresponding authors: aramamoorthy@fsu.edu & bsahoo@umich.edu

**Supplementary contents:**

Supporting methods S1-S4

Supporting Figures (S1-S6) S5-S10

Supporting videos SV1-SV11 S11

## Supplementary methods

**Chemicals**: 1,2-dimyristoyl-sn-glycero-3- phosphatidylcholine (DMPC) and 1,2-dimyristoyl-sn-glycero-3-phosphorylglycerol (DMPG) lipids were purchased from Avanti Polar Lipids, Inc® (Alabaster, AL). All other reagents used in this study were purchased from Sigma-Aldrich®.

**Peptide and Polymer Sample Preparation**: Human-IAPP (hIAPP) peptides (KCNTATCATQRLANFLVHSSNNFGAILSSTNVGSNTY-$NH_2$) were purchased from AnaSpec (purity >95%). The hIAPP sample preparation method is reported in our previous study.[1] Briefly, 1 mg/mL hIAPP was suspended in 1,1,1,3,3,3-hexafluoroisopropanol (HFIP), and incubated on ice for ~ 30 minutes. Samples were aliquoted into 0.1 mg/mL, lyophilized for ~48 hours, and resuspended in 30 mM sodium acetate (NaOAc) at pH 5.5 on ice at a final concentration of 50 µM. The solution was sonicated for ~15 seconds, and centrifuged at 14,000 rpm for 2 minutes to remove any aggregates. The resultant hIAPP monomers were immediately used in experiments or flash-frozen and stored at -80 °C. Uniformly $^{15}$N-labeled hIAPP peptides were recombinantly expressed and purified as reported elsewhere.[1] The concentration of hIAPP was spectroscopically measured using a nanodrop and peptide extinction coefficient of 1490 $M^{-1}$ $cm^{-2}$.

The 4F (Ac-DWFKAFYDKVAEKFKEAF-NH2) peptide was synthesized using solid-phase peptide synthesis and purified by HPLC as reported elsewhere[3]. The polymethacrylate copolymer (PMA) was synthesized and purified at the Nara Institute of Technology, Japan as reported elsewhere[4] (a gift from Professor Kazuma Yasuhara).

**Nanodisc Preparation**: The nanodisc preparation protocol was adopted from our previous studies.[4–6] Briefly, 4F or PMAQA solid powder was dissolved in 30 mM NaOAc, 50 mM NaCl, pH 7.4 at 8 mg/mL (for 4F) or 4 mg/mL (for PMAQA), and mixed with 2 mg/mL of 4:1 DMPC:DMPG molar ratio. The DMPC/DMPG lipid mixture was first prepared in a glass tube by dissolving the lipid powders obtained from Avanti Polar Lipids in 100% chloroform. The organic solvent was evaporated by gently passing nitrogen gas to form DMPC/DMPG lipid film. The lipid film was next resuspended in 1 mL 30 mM NaOAc, 50 mM NaCl, pH 7.4. Next, 0.5 mL of lipids were mixed with 0.5 mL of 8 mg/mL 4F peptides or 4 mg/mL PMAQA polymers. Samples were vortexed every 15 minutes for the first 3 hours and incubated at 37 °C overnight for nanodiscs formation. Nanodiscs formation was verified by observing the transition of an opaque solution to a transparent solution and their sizes were tested using dynamic light scattering (DLS) measurements. The nanodiscs were purified using size-exclusion chromatography (SEC) in 30 mM NaOAc, pH 5.5. The purified nanodisc concentration, monodispersity, and size distribution were further accessed following previously reported protocols.[4,7]

**Thioflavin-T Fluorescence Assay**: The aggregation kinetics of hIAPP was studied using Thioflavin T (ThT) dye-based fluorescence assay. The aggregation of freshly dissolved 5 µM hIAPP was monitored at 37 °C in the absence and presence of nanodiscs. The aggregation assay was carried out on a Fisher low-binding 96-well plate. A sample mixture of 100 µL/well containing 5 µM peptide, variable nanodisc concentration, and 10 µM ThT was prepared in replicates. Fluorescence intensity was recorded every 3 minutes for 3 hours using a Biotek Synergy 2 microplate reader until a fibril growth saturation was achieved for hIAPP samples containing no nanodiscs. The excitation and emission wavelengths of 440 and 485 nm, respectively, were used for ThT fluorescence measurements.

**Circular Dichroism (CD) Spectroscopy**: Changes in the secondary structure of hIAPP were monitored by performing CD experiments on a JASCO - J820 spectropolarimeter. Far-UV CD spectra (190-260 nm) of 20 μM hIAPP dissolved in 30 mM NaOAc, pH 5.5 mixed without or with PMA-nanodiscs were recorded at 30 °C at different time intervals (15 minutes to 168 hours). The buffer CD signals were subtracted from the peptide signals in all CD measurements and the mean residue ellipticity [Θ] was plotted by averaging data from 8 scans.

**Dynamic Light Scattering (DLS)**: The monodispersity and size-distribution of 4F- and PMA-nanodiscs in the absence and presence of hIAPP at different time intervals were studied using DLS measurements (Wyatt Technology Corporation, Goleta, CA) at 37 °C. Nanodiscs were incubated with 5 μM hIAPP (1:20 hIAPP:nanodiscs) in 30 mM NaOAc, pH 5.5 at 37 °C for DLS measurements. DLS measurements averaged over 10 acquisitions and the percentage mass of nanodiscs was plotted against the hydrodynamic radius by fitting the data to an isotropic sphere model using the in-built Wyatt Technology instrument software.

**High-speed Atomic Force Microscopy**: hIAPP aggregation in real-time was monitored using an in-house built high-speed atomic force microscopy (HS-AFM) instrument (at Kanazawa University, Japan). The method used for HS-AFM imaging was adopted from a previous study.[8] Briefly, the instrument was equipped with a small cantilever (BL-AC10DS-A2, Olympus) with an amorphous carbon tip operated in tapping mode with a spring constant k = 0.1 N $m^{-1}$, and resonance frequency f = 400 kHz in water. A freshly cleaved 1-mm diameter mica disc was fixed on a 2-mm diameter glass rod to prepare for the HS-AFM stage. Variable concentrations of nanodiscs, hIAPP, or hIAPP mixed with nanodiscs (~60 μL) were deposited on the mica surface. Unbound sample particles were removed by washing the mica stage using 30 mM NaOAc buffer, pH 5.5. For HS-AFM studies to monitor the growth of hIAPP fibrils, fibers were formed by incubating 40 μM hIAPP monomers for 4 days mixed with or without equal amounts of PMA- or 4F-nanodiscs for 24 hours at room temperature. The samples were next precipitated by spinning at 20,000 × g for 30 minutes. The precipitated samples were next resuspended in 30 mM NaOAc, pH 5.5, and deposited on the HS-AFM stage for imaging. The mica surface was washed with 60 μL of 30 mM NaOAc, pH 5.5, to remove any unbound particles. The fibril seeds deposited on the mica surface were first observed, following which the chamber buffer solution was replaced with freshly dissolved hIAPP monomers to achieve the desired concentration in the solution chamber, and fibril growth was monitored immediately. The HS-AFM images and movies were analyzed using ImageJ (NIH).

**Nuclear Magnetic Resonance (NMR) Spectroscopy**: The interaction of hIAPP with nanodiscs was probed using NMR spectroscopy. 1D proton NMR spectra of 40 μM hIAPP peptides freshly dissolved in 30 mM sodium acetate, pH 5.5, 10% $D_2O$ mixed without or with nanodiscs containing 250 μM lipids, were acquired with 512 scans and a 2 s delay at 25 °C. 2D SOFAST-$^{1}H/^{15}N$ HMQC NMR spectra were recorded for 30 μM hIAPP freshly dissolved in 30 mM sodium acetate, pH 5.5, 10% $D_2O$ mixed without or with 250 μM lipids using 16 scans, 256 t1 increments, and a 0.2 s recycle delay at 25 °C. NMR spectra were recorded ~1 hr after sample preparation in the order hIAPP, hIAPP+4F nanodiscs, and hIAPP+PMA nanodiscs. The NMR data collection for one sample took us about ~0.5 hours. 2D All 1D NMR spectra were collected on a 500 MHz Bruker NMR spectrometer equipped with a z-axis gradient triple-resonance (TXO) probe, and 2D spectra were recorded on an 800 MHz Bruker spectrometer equipped with a triple-resonance cryoprobe. All NMR spectra were processed using TopSpin 3.5 (Bruker) and analyzed using NMRFAM Sparky.[9]

**Coarse-grained molecular dynamics (MD) simulations**: Coarse-grained (CG) models of hIAPP and nanodisc containing DLPC lipids were designed using the Martini force field[10] framework to study the interaction between hIAPP and nanodiscs. The 4F nanodiscs were self-assembled by simulating 20 4F peptides and 80 DLPC lipids for 10 μs in the Martini force field using the Gromacs 5.0.7 program.[11] The CG model of PMA-nanodisc of size ~8 nm, previously optimized and tested for protein reconstitution, and self-assembled 4F nanodisc was used to study the interaction of two hIAPP molecules placed > 1 nm away from the bilayer surface using Gromacs. The hIAPP and nanodisc CG systems were designed using our previous method and a final production MD run was set for 5 μs at 310 K. The MD snapshots were retrieved at different time points from the 5 μs trajectory to track the hIAPP binding to nanodiscs. The CG simulations were run on SGI UV 3000 in parallel using a high-performance computing facility at the Institute for Protein Research, Osaka University, Japan.

## References


1. D. C. Rodriguez Camargo, K. Tripsianes, T. G. Kapp, J. Mendes, J. Schubert, B. Cordes and B. Reif, *Protein. Expr. Purif.*, 2015, **106**, 49–56.
2. B. R. Sahoo, T. Genjo, T. W. Nakayama, A. K. Stoddard, T. Ando, K. Yasuhara, C. A. Fierke and A. Ramamoorthy, *Chem. Sci.*, 2019, **10**, 3976–3986.
3. G. Datta, M. Chaddha, S. Hama, M. Navab, A. M. Fogelman, D. W. Garber, V. K. Mishra, R. M. Epand, R. F. Epand, S. Lund-Katz, M. C. Phillips, J. P. Segrest and G. M. Anantharamaiah, *J. Lipid Res.*, 2001, **42**, 1096–1104.
4. K. Yasuhara, J. Arakida, T. Ravula, S. K. Ramadugu, B. Sahoo, J. I. Kikuchi and A. Ramamoorthy, *J. Am. Chem. Soc.*, 2017, **139**, 18657–18663.
5. B. R. Sahoo, T. Genjo, S. J. Cox, A. K. Stoddard, G. M. Anantharamaiah, C. Fierke and A. Ramamoorthy, *J. Mol. Biol.*, 2018, **430**, 4230–4244.
6. B. R. Sahoo, T. Genjo, M. Bekier, S. J. Cox, A. K. Stoddard, M. Ivanova, K. Yasuhara, C. A. Fierke, Y. Wang and A. Ramamoorthy, *Chem. Commun.*, 2018, **54**, 12883–12886.
7. B. R. Sahoo, T. Genjo, S. J. Cox, A. K. Stoddard, G. M. Anantharamaiah, C. Fierke and A. Ramamoorthy, *J. Mol. Biol.*, 2018, **430**, 4230–4244.
8. T. Watanabe-Nakayama and K. Ono, High-speed atomic force microscopy of individual amyloidogenic protein assemblies, Methods Mol. Biol. 2018, **1814**, 201-212.
9. W. Lee, M. Tonelli and J. L. Markley, Bioinformatics, 2015, **31**, 1325-1327.
10. S. J. Marrink, H. J. Risselada, S. Yefimov, D. P. Tieleman and A. H. De Vries, J. Phys.Chem. B, 2007, **111**, 7812–7824.
11. D. Van Der Spoel, E. Lindahl, B. Hess, G. Groenhof, A. E. Mark and H. J. C. Berendsen, J. Comput. Chem., 2005, **26**, 1701–1718.

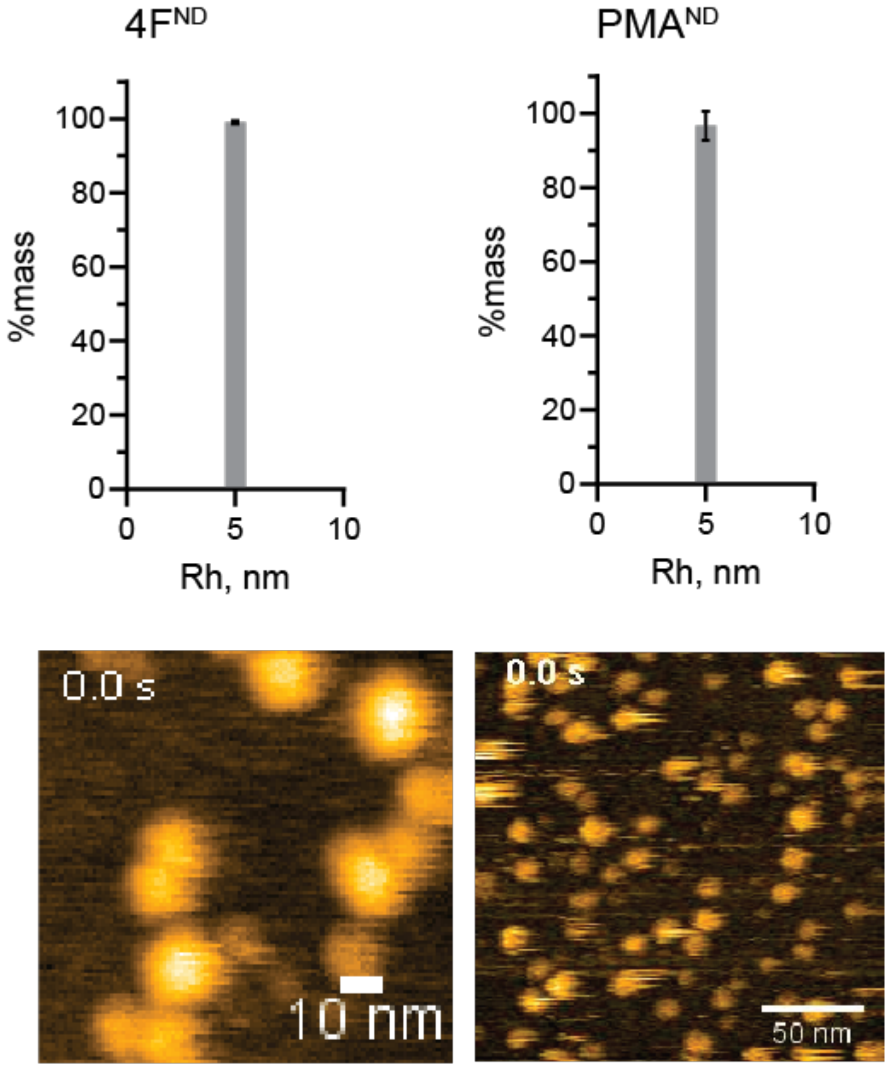


**Figure S1. Size distribution analysis of nanodiscs measured by DLS and HS-AFM.** Hydrodynamic radius (Rh) of 4F- (top left) and PMA-nanodiscs (top right) containing 20 μM 4:1 DMPC:DMPG molar ratio. The standard deviation presents data averaging from 10 acquisitions. HS-AFM images of 4:1 DMPC:DMPG 4F-nanodiscs (bottom left) and PMA-nanodiscs (bottom right).

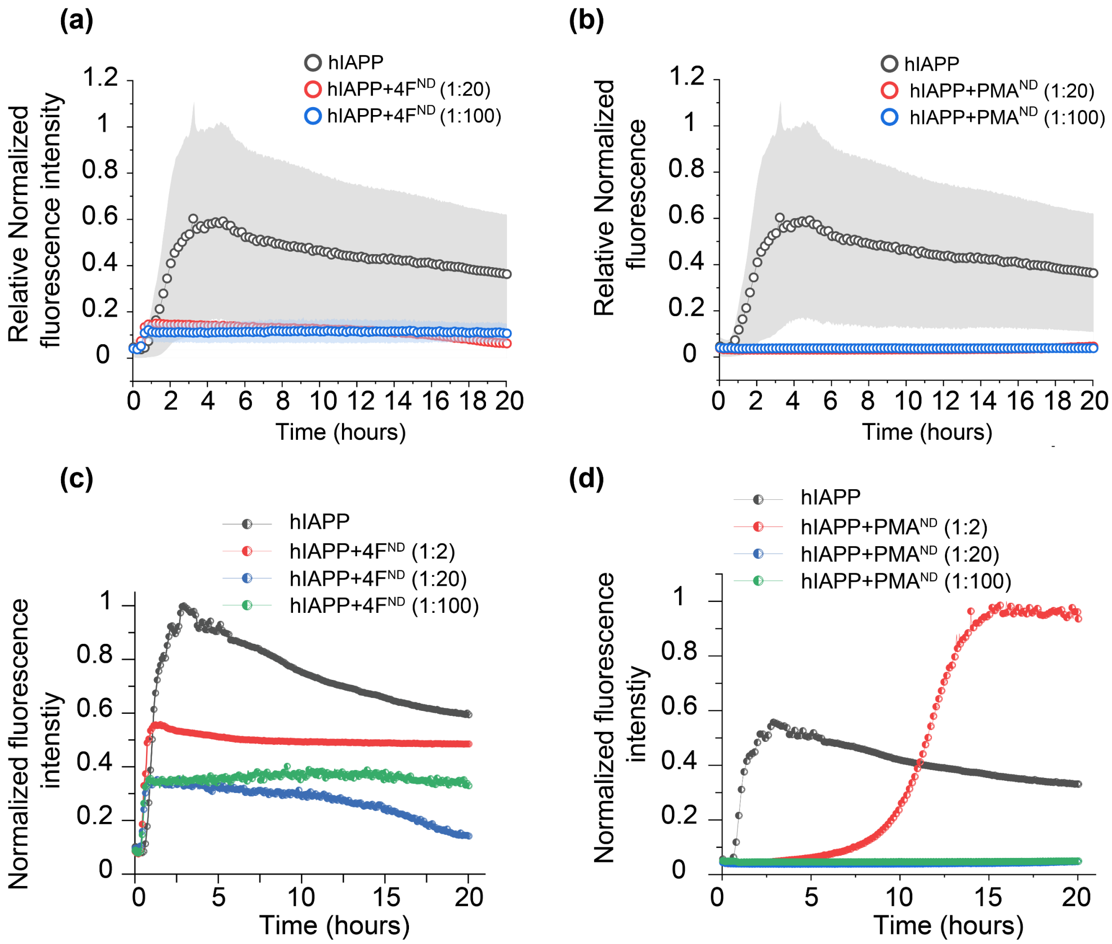


**Figure S2.** Enlarged regions of Figure 1(b and c) showing the aggregation kinetics of hIAPP monitored using ThT fluorescence for 20 hours. Normalized ThT fluorescence aggregation of 5 μM hIAPP dissolved in 20 mM NaOAc, pH 5.5 at 37 °C mixed with 4F-nanodiscs (a) and PMA-nanodiscs (b) at the indicated peptide:lipid molar ratios relative to hIAPP in the absence of nanodiscs. (c-d) Since the ThT fluorescence kinetics curves obtained in the presence of nanodiscs are overlapped in (a) and (b), they are plotted by normalizing the fluorescence intensity relative to the maximum yield obtained in each set of experiments containing either 4F (c) or PMA (d) nanodiscs. Results obtained in presence of 2 molar nanodiscs are also plotted in (c) and (d).

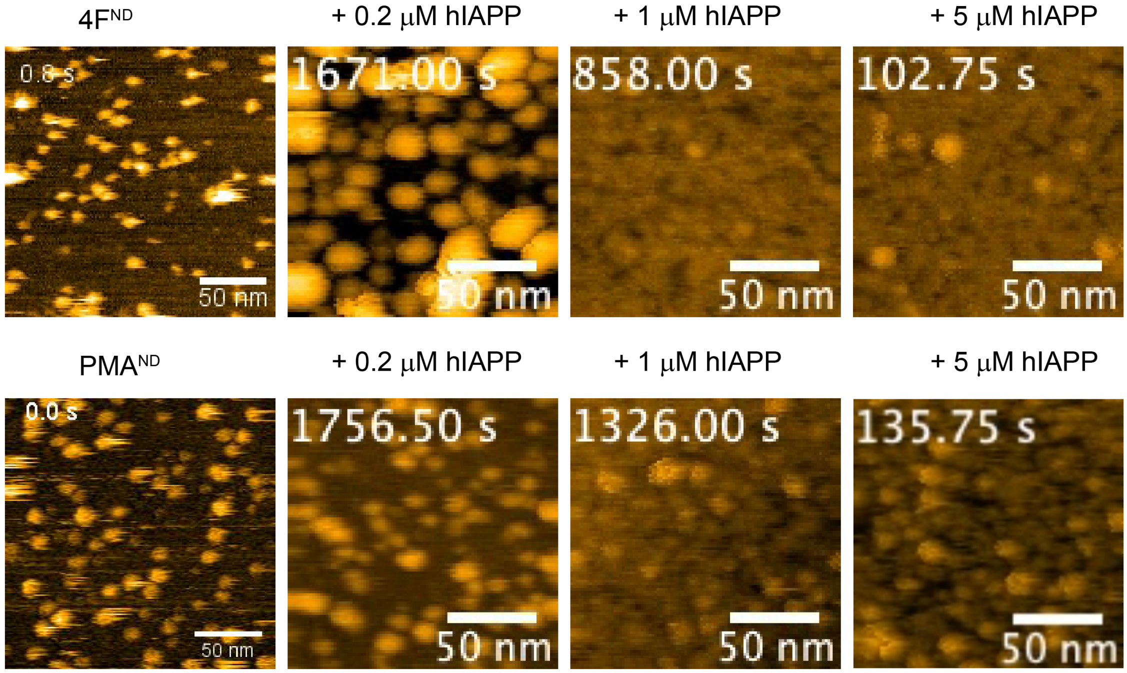


**Figure S3. HS-AFM images show hIAPP molecules fill within the nanodiscs on the mica surface.** The hIAPP, 4F-, and PMA-nanodiscs samples were prepared in 30 mM NaOAc, pH 5.5, and HS-AFM videos were recorded at room temperature. The corresponding HS-AFM videos are SV1, SV2, SV3, SV4, SV5, SV6, SV7 and SV8.

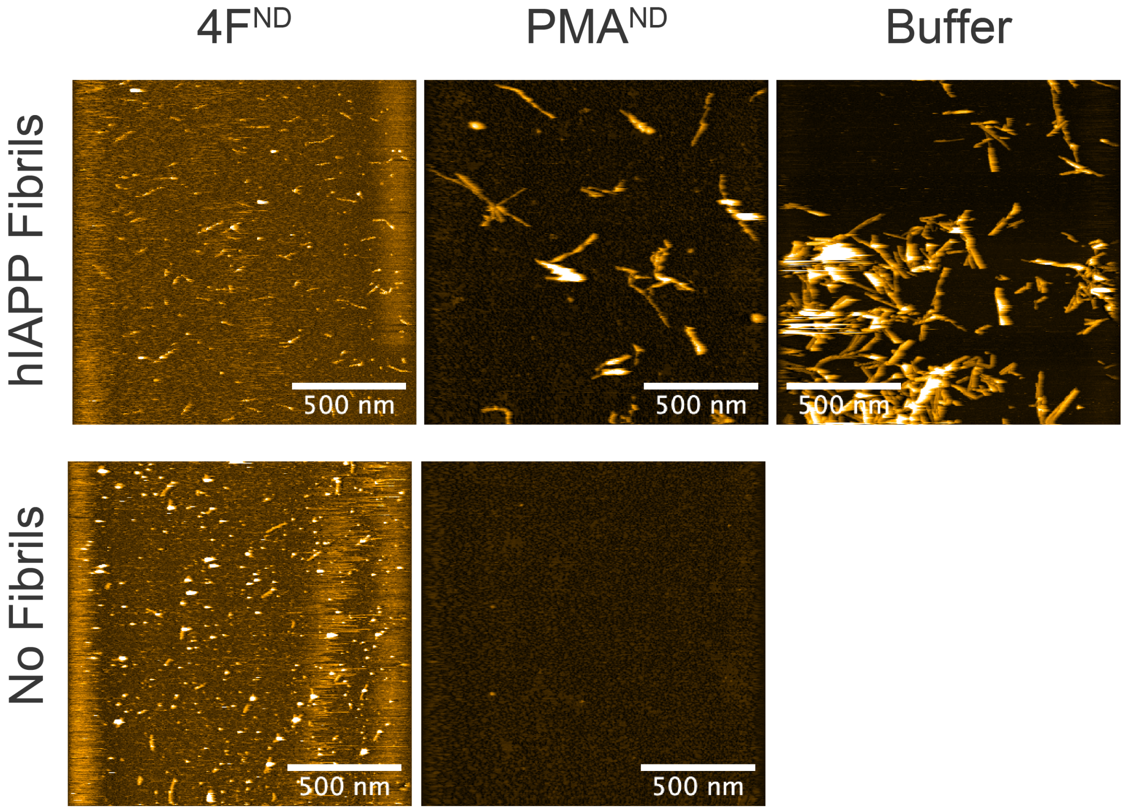


**Figure S4. Dilution of the excess nanodiscs enhanced the hAIPP fibril binding to mica in PMA-nanodiscs, but not in 4F-nanodiscs.** Sonicated 20 μM fibrils and nanodiscs suspended in 30 mM NaOAc, pH 5.5 (see methods for sample preparation) were deposited on mica, and unbound samples were washed out before HS-AFM imaging.

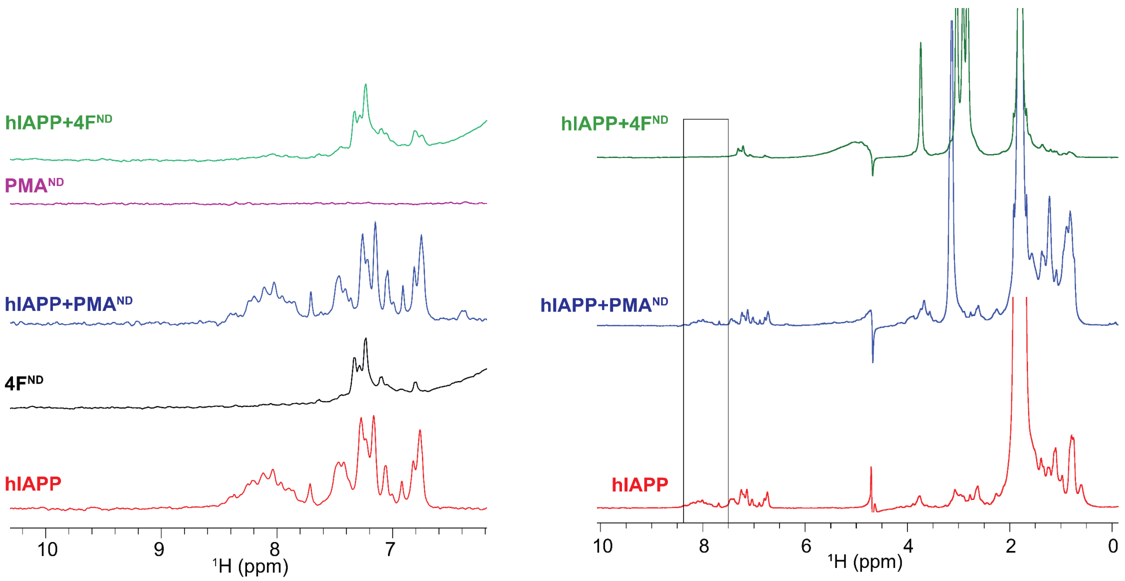


**Figure S5. 1D proton NMR spectra of hIAPP mixed with or without nanodiscs.** The left panel shows non-overlapped NMR spectra of the amid protons that correspond to the overlapped proton spectra shown in Figure 1g. NMR samples were prepared by dissolving 40 µM hIAPP in 30 mM NaOAc, pH 5.5, in the absence (red spectrum) or presence of different nanodiscs. The right panel shows the full 1D NMR spectrum of hIAPP mixed without or with nanodiscs, which corresponds to the zoom amide-region spectra shown on the left panel or in Figure 1g.

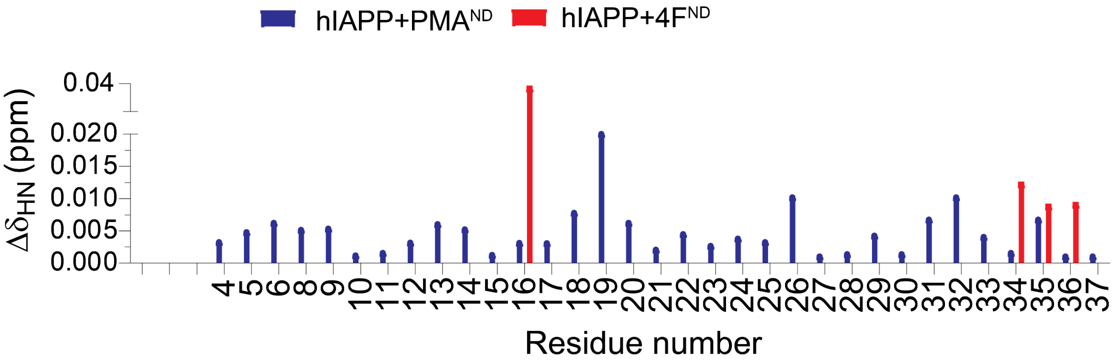


**Figure S6. Residue-specific analysis of hIAPP binding to nanodiscs.** Residue-wise chemical shift perturbations for hIAPP upon binding PMA (blue) and 4F nanodiscs (red). The corresponding NMR spectra are shown in main text Figure 3. The hIAPP peptides were dissolved in 30 mM NaOAc at pH 5.5, and NMR spectra were acquired at 30 °C using a Bruker 800 MHz spectrometer. Chemical shift perturbations were computed using the equation $\Delta\delta_{NH} = \sqrt{(\delta^1H)^2 + 0.154 \times (\delta^{15}N)^2}$.

**Supporting videos**

**Supporting video SV1**: HS-AFM video of 4F nanodiscs containing 10 nM of 4:1 DMPC:DMPG molar ratio recorded at room temperature. The samples were prepared in 30 mM NaOAc buffer, pH 5.5.

**Supporting video SV2**: HS-AFM video of PMA nanodiscs containing 20 μM of 4:1 DMPC:DMPG molar ratio recorded at room temperature. The samples were prepared in 30 mM NaOAc buffer, pH 5.5.

**Supporting video SV3**: HS-AFM video of 4F nanodiscs containing 10 μM of 4:1 DMPC:DMPG molar ratio followed by addition of 0.2 μM hIAPP at 64 s at room temperature. The samples were prepared in 30 mM NaOAc buffer, pH 5.5.

**Supporting video SV4**: HS-AFM video of 4F nanodiscs containing 10 μM of 4:1 DMPC:DMPG molar ratio followed by addition of 1 μM hIAPP at 66 s at room temperature. The samples were prepared in 30 mM NaOAc buffer, pH 5.5.

**Supporting video SV5**: HS-AFM video of 4F nanodiscs containing 10 μM of 4:1 DMPC:DMPG molar ratio followed by addition of 5 μM hIAPP at 28 s at room temperature. The samples were prepared in 30 mM NaOAc buffer, pH 5.5.

**Supporting video SV6**: HS-AFM video of PMA nanodiscs containing 50 nM of 4:1 DMPC:DMPG molar ratio followed by addition of 0.2 μM hIAPP at 25 s at room temperature. The samples were prepared in 30 mM NaOAc buffer, pH 5.5.

**Supporting video SV7**: HS-AFM video of PMA nanodiscs containing 50 nM of 4:1 DMPC:DMPG molar ratio followed by addition of 1 μM hIAPP at 32 s at room temperature. The samples were prepared in 30 mM NaOAc buffer, pH 5.5.

**Supporting video SV8**: HS-AFM video of PMA nanodiscs containing 50 nM of 4:1 DMPC:DMPG molar ratio followed by addition of 5 μM hIAPP at 54 s at room temperature. The samples were prepared in 30 mM NaOAc buffer, pH 5.5.

**Supporting video SV9**: HS-AFM movie of the centrifuged pellet from the reaction solution after incubating 20 μM hIAPP fiber at room temperature for 1 day in the absence of nanodiscs, followed by the addition of 5 μM hIAPP monomer for 1 minute.

**Supporting video SV10**: HS-AFM movie of the centrifuged pellet from the reaction solution after incubating 20 μM hIAPP fiber at room temperature for 1 day in the presence of 4F nanodiscs containing 20 μM of lipids in 30 mM NaOAc buffer, pH 5.5, followed by the addition of 5 μM hIAPP monomer for 1 minute in the video. The fibers that appear and grow at the end of the video are de novo fibrils formed from IAPP monomer in the chamber.

**Supporting video SV11**: HS-AFM movie of the centrifuged pellet from the reaction solution after incubating 20 μM hIAPP fiber at room temperature for 1 day in the presence of PMA nanodiscs containing 20 μM of lipids in 30 mM NaOAc buffer, pH 5.5, followed by the addition of 5 μM hIAPP monomer for 1 minute in the video.
